# Complete Genome Sequences of Two *Listeria* Phages of the Genus *Pecentumvirus*

**DOI:** 10.1128/MRA.01229-19

**Published:** 2019-11-14

**Authors:** Tracey L. Peters, Lauren K. Hudson, Yaxiong Song, Thomas G. Denes

**Affiliations:** a Department of Food Science, University of Tennessee, Knoxville, Tennessee, USA; Queens College

## Abstract

Bacteriophages isolated from environmental sources can be used as a biocontrol against the foodborne pathogen Listeria monocytogenes. Here, we present the complete genomes of LP-039 and LP-066, two *Pecentumvirus* bacteriophages that infect L. monocytogenes. The genome sizes of LP-039 and LP-066 are 136.2 kb and 139.0 kb, respectively.

## ANNOUNCEMENT

Listeria monocytogenes has caused 1,151 infections in the United States between 2010 and 2018, with a 17% mortality rate (1), and was mainly associated with contaminated dairy or fruit products (2). Lytic *Listeria* phages, such as *Pecentumvirus* P100, are used to control L. monocytogenes in the food industry (3–7). Phages LP-039 and LP-066 are from a collection of *Listeria* phages previously isolated from silage samples obtained from New York dairy farms using L. monocytogenes strain MACK as previously described (8). These phages are of interest because they exhibit activity against two phage-resistant L. monocytogenes strains (9).

Phage DNA was isolated using a phenol-chloroform method (10), and libraries were prepared using Nextera XT kits. Samples were sequenced with an Illumina MiSeq v3 instrument (300-bp paired-end read chemistry; 275 cycles). Total read numbers of 55,642 and 160,826 were obtained for LP-039 and LP-066, respectively. The average read length was 251 bp. Reads were preprocessed with Trimmomatic v0.35 (ILLUMINACLIP:NexteraPE-PE.fa:2:30:10 LEADING:3 TRAILING:3 SLIDINGWINDOW:4:15 MINLEN:36) (11) and FastQC v0.11.7 (12). Single contigs were assembled using SPAdes v3.12.0 with the careful option (13). The redundant terminal region of A511 (14) mapped to the contigs at internal loci with higher read coverage (∼2× greater) than the rest of the contig. The contigs were reoriented so that these redundant terminal regions were correctly located at both ends. This was confirmed by mapping reads to the reoriented contigs to ensure that coverage across the redundant terminal regions and the newly formed contig junctions (where original contig ends were joined) was consistent with the rest of the assembly. Assembly statistics were determined using QUAST v4.6.3 (15), BBMap v38.88 (16), and SAMtools v0.1.8 (17). The genomes were annotated with RASTtk (customized pipeline, “annotate-proteins-phage” moved above “annotate-proteins-kmer-v2”) (18), and annotations of the redundant terminal regions were manually added. Relatedness to *Pecentumvirus* phages (ICTV Master Species List 2018b.v2 [https://talk.ictvonline.org/files/master-species-lists/m/msl/8266]) was determined with the JSpeciesWS average nucleotide identity MUMmer (ANIm) method (19). Variant analysis was performed with McCortex v0.0.3 (20) (k = 101; breakpoint caller; JOINT_CALLING=yes; USE_LINKS=yes) and SnpEff v4.3t (21).

LP-039 and LP-066 have terminally redundant linear genomes with large invariable, noncohesive ends. LP-039 had a total genome assembly length of 136,234 bp (including the 3,208-bp terminal redundancy) with 88× average coverage and 35.9% G+C content. LP-066 had a total genome assembly length of 138,918 bp (including the 3,128-bp terminal redundancy) with 272× average coverage and 35.8% G+C content. Both genomes contained 193 to 198 coding sequences (each contained 9 duplicate coding sequences due to the terminal redundancy) and 17 tRNAs. LP-039 is closely related to LP-048 (Table 1). Variant analysis of LP-039 compared to LP-048 showed one mutation (a 3-nucleotide deletion) in LP048_062 (hypothetical protein) with potential upstream effects in tRNA genes. LP-066 is closely related to LP-083-2 (Table 1). Variant analysis of LP-066 compared to LP-083-2 showed four mutations. LP083-2_021 and LP083-2_130 (hypothetical proteins) each had one synonymous mutation with minimal predicted effects. One conservative nonsynonymous mutation (G > T) was found at position 1871 in gene LP083-2_152 (DNA polymerase I), producing a valine rather than a glycine. A 15-nucleotide in-frame insertion was found in gene LP083-2_101 (hypothetical protein) near genes involved in recombination. This mutation resulted in the duplication of the amino acid sequence K-E-E-P-K.

**TABLE 1:** Results from JSpeciesWS for *Pecentumvirus Listeria* phages

Phage	Avg nucleotide identity (%) shared between phage pairs^*a*^												
	A511	AG20	List36	LMSP25	LMTA34	LMTA148	LP-048	LP-064	LP-083-2	P100^*b*^	WIL1	LP-039	LP-066
A511		97.14 (97.12)	97.09 (95.00)	96.39 (95.67)	96.39 (95.67)	97.24 (95.51)	97.20 (96.02)	97.15 (97.57)	97.00 (97.84)	97.20 (95.13)	97.45 (98.52)	97.14 (96.03)	96.96 (97.84)
AG20	97.14 (98.43)		97.38 (96.29)	96.30 (96.46)	96.30 (96.46)	97.60 (96.36)	97.50 (98.15)	97.18 (98.39)	97.15 (98.36)	97.14 (96.39)	97.20 (98.00)	97.50 (98.16)	97.14 (98.36)
List36	97.09 (97.05)	97.41 (96.93)		96.56 (96.18)	96.56 (96.18)	97.60 (96.48)	97.41 (97.54)	97.23 (98.24)	97.40 (98.03)	97.16 (97.28)	97.26 (96.72)	97.40 (97.54)	97.40 (98.03)
LMSP25	96.39 (94.25)	96.30 (93.74)	96.56 (92.85)		100.00 (100.00)	96.78 (91.46)	96.42 (92.67)	96.64 (95.30)	96.57 (95.60)	96.71 (93.10)	96.40 (93.96)	96.41 (92.74)	96.59 (95.60)
LMTA34	96.39 (94.25)	96.30 (93.74)	96.56 (92.85)	100.00 (100.00)		96.78 (91.46)	96.42 (92.67)	96.64 (95.30)	96.57 (95.60)	96.71 (93.10)	96.40 (93.96)	96.41 (92.74)	96.59 (95.60)
LMTA148	97.24 (97.25)	97.60 (96.48)	97.60 (96.09)	96.78 (94.32)	96.78 (94.32)		97.69 (98.04)	97.15 (97.01)	97.28 (96.71)	97.28 (95.18)	97.17 (97.07)	97.67 (98.04)	97.26 (96.72)
LP-048	97.20 (97.78)	97.50 (98.13)	97.41 (96.83)	96.42 (95.36)	96.42 (95.36)	97.69 (97.87)		97.14 (98.29)	97.11 (98.30)	97.01 (96.71)	97.28 (97.58)	99.99 (100.00)	97.14 (98.30)
LP0-64	97.15 (97.22)	97.18 (96.76)	97.25 (95.88)	96.64 (96.49)	96.64 (96.49)	97.15 (95.22)	97.18 (96.73)		98.13 (99.05)	98.20 (97.22)	97.33 (97.14)	97.14 (96.74)	98.10 (99.06)
LP-083-2	97.00 (97.05)	97.15 (96.33)	97.37 (95.33)	96.57 (96.33)	96.57 (96.33)	97.28 (94.53)	97.11 (96.27)	98.13 (98.62)		98.32 (96.26)	97.35 (97.12)	97.11 (96.29)	99.98 (100.00)
P100^*b*^	97.20 (97.46)	97.14 (97.49)	97.16 (97.68)	96.71 (96.98)	96.71 (96.98)	97.20 (96.43)	97.01 (97.86)	98.19 (99.97)	98.32 (99.43)		97.34 (97.89)	97.01 (97.86)	98.33 (99.41)
WIL1	97.42 (98.63)	97.22 (96.89)	97.26 (95.11)	96.40 (95.50)	96.40 (95.50)	97.17 (95.73)	97.28 (96.41)	97.33 (97.78)	97.35 (98.07)	97.34 (95.73)		97.28 (96.41)	97.33 (98.07)
LP-039	97.14 (97.31)	97.50 (97.98)	97.40 (96.91)	96.41 (95.15)	96.41 (95.15)	97.67 (97.92)	99.99 (100.00)	97.14 (98.14)	97.11 (98.21)	97.01 (96.58)	97.28 (97.23)		97.14 (98.21)
LP-066	96.96 (97.03)	97.14 (96.46)	97.37 (95.48)	96.59 (96.34)	96.59 (96.34)	97.26 (94.60)	97.14 (96.29)	98.10 (98.68)	99.98 (100.00)	98.33 (96.38)	97.33 (97.23)	97.14 (96.31)	

a Numbers represent the average nucleotide identity across aligned nucleotide percentage (aligned percentage is in brackets).

b Type species of the genus *Pecentumvirus*.

LP-039 and LP-066 were independently isolated from similar environmental sources as *Listeria* phages LP-048 and LP-083-2 (22) and are likely examples of recent evolutionary genetic divergence from a common ancestor under natural conditions. Genomic characterization of closely related phages such as the ones presented here will provide valuable information on genetic variation between wild phage strains and may help identify candidate phages for biocontrol applications.

### Data availability.

These phages are located under BioProject number PRJNA544516 (BioSample numbers SAMN12053438 and SAMN12053439). The raw reads have been deposited in the SRA (accession numbers SRR9597082 and SRR9597083), and the annotated genomes in GenBank (accession numbers MN172529 and MN128594).
